# Atypical Porcine Pestivirus as a Novel Type of Pestivirus in Pigs in China

**DOI:** 10.3389/fmicb.2017.00862

**Published:** 2017-05-11

**Authors:** Jin Yuan, Zhiyong Han, Jun Li, Yunzhen Huang, Jiongfeng Yang, Hongxing Ding, Jingyuan Zhang, Mengjiao Zhu, Yangyi Zhang, Jiedan Liao, Mingqiu Zhao, Jinding Chen

**Affiliations:** College of Veterinary Medicine, South China Agricultural UniversityGuangzhou, China

**Keywords:** congenital tremor, APPV, China, tissue tropism, sequence analysis

## Abstract

Pestiviruses are highly variable RNA viruses. A growing number of novel pestiviruses has been discovered in domestic and wild species in the last two decades. Recently, a novel atypical porcine pestivirus (APPV) linked with the development of congenital tremor (CT) in neonatal pigs was described in Europe and the Americas. Here, the first Asian APPV complete polyprotein coding sequence was assembled from serum samples from newborn piglets affected with CT in Southern China, and termed APPV_GD. 14 organ samples from affected piglets were analyzed by quantitative RT-PCR (qRT-PCR) to investigate the tissue tropism of APPV, and 135 serum samples from pigs from 10 farms were used for identifying APPV in adult pigs. The highest genome loads were found in submaxillary lymph nodes, and PCR-based detection showed that APPV genomes were present in seven samples from five farms. A phylogenetic tree was constructed based on the full-length genomes of the pestiviruses, and APPV_GD appeared on a new branch with another newly discovered APPV. Nucleotide identity analysis demonstrated that APPV_GD shared the highest nucleotide sequence identity with a German APPV. Bayesian inference was performed using 25 partial sequences of the APPV NS5B gene (528 bp) isolated from four countries in recent years. According to this analysis, the most recent common ancestor (tMRCA) of the current APPV strains might have emerged in Germany and then diversified and spread to Asia, the Americas, and other countries in Europe. However, the result of bayesian inference could change when more APPV strains are isolated in the future. The present study is the first to report APPV in China and infers the origin and dissemination of the current strains of the virus.

## Introduction

Pestiviruses are highly variable RNA viruses that cause huge economic losses in the global pig breeding industry. In recent years, several novel pestiviruses have been discovered in domestic and wild species, including a broad spectrum of ruminant pestiviruses termed Bungowannah (Kirkland et al., 2007), a Norwegian rat pestivirus known as NrPV (Firth et al., 2014), and a putative pestivirus isolated from bats, which is known as RaPV (Wu et al., 2012). Hause et al. (2015) found a novel pestivirus, namely the atypical porcine pestivirus (APPV), in porcine serum via metagenomic sequencing. Soon thereafter, sera from APPV-infected pigs were shown to cause congenital tremor (CT) (Arruda et al., 2016), and this study was the first report regarding reproduction of CT in an animal model. Three other studies published during this period suggested that the presence of APPV genomes in newborn piglets was correlated with CT (de Groof et al., 2016; Postel et al., 2016; Schwarz et al., 2017).

Congenital tremor is a disease of neonatal pigs that is characterized by local or systemic muscle spasms observed within hours of birth. In piglets, mild symptoms only appear as evident tremors in the ears, flank, or hind leg region. Severe symptoms include systemic tremors that result in difficulty in standing or walking and the consequent inability to nurse, leading to the death of most infected piglets from starvation. Furthermore, the few surviving piglets are euthanized, as their prognosis is poor.

In China, CT was first reported in the 1960s. Over the subsequent few decades, CT was reported in most parts of the country. The 100% elimination of infected piglets resulted in huge economic losses to the pig industry. Recent studies have suggested a positive link between APPV and CT; however, no studies of APPV in China have been reported to date. The present study aimed to determine whether APPV is present in China. Here, we assembled the first Asian APPV complete polyprotein coding sequence, and performed bioinformatics analyses of current APPV strains.

## Materials and Methods

### Sample Collection

Diagnostic tests were performed at our laboratory on three 3-day-old piglets with CT from two farms in southern China. Serum, heart, liver, spleen, lungs, kidneys, brain, cerebellum, brainstem, inguinal lymph nodes, submaxillary lymph nodes, tonsils, spinal cord, duodenum, and bladder were collected from the three CT-affected piglets at the age of one week. This study was carried out in accordance with animal ethics guidelines and approved by the Animal Care and Use Committee of South China Agriculture University, China. The issue number is 2016-07. In addition, 135 randomly selected serum samples from sows (8 to 41 months) and fattening pigs (25 to 170 days) originating from 10 farms were obtained from the Guangdong Academy of Agricultural Sciences. These serum samples were used for veterinary microbiological diagnosis, and residual volumes were provided for use in the present study.

### RNA and cDNA Preparation

Viral RNA was isolated from serum and various organs using the Viral RNA Kit according to the manufacturer’s protocol (OMEGA Bio Inc. USA, R6874-02), and three independent pieces (25 mg) of each organ sample collected from one of the three CT-affected piglets were tested. Reverse transcription was performed using the Random Primer 6N (Takara Bio Inc. Dalian, 3801) and the PrimeScript RT Master Mix (RR036A; Takara Bio Inc. Dalian, RR036A) according to the manufacturer’s protocol.

### Reverse Transcription PCR (RT-PCR)

To perform APPV detection and screening, PCR primers for NS3 and NS5B were designed based on published sequences as previously reported (Postel et al., 2016) (**Table 1**). The NS5B primer (APPV_NS5B-F/APPV_NS5B-R) was used for APPV screening by PCR using Premix Taq (Takara Bio Inc. Dalian, R004Q). The NS3 primer (APPV_NS3-F/APPV_NS3-R) was used for quantitative RT-PCR (qRT-PCR) to determine the tissue tropism of APPV. For preparing the RNA standard, the T7 promoter was added to the 5′ end of the NS3 forward primer as another forward primer named APPV_NS3-T7-F (**Table 1**). The primer (APPV_NS3-T7-F/APPV_NS3-R) was used f fragment amplification, and the PCR products were used as a template for *in vitro* transcription. The transcription was performed using the *In vitro* Transcription T7 Kit (Takara Bio Inc. Dalian, 6140) according to the manufacturer’s protocol. The concentration and the OD value of the RNA standards were determined. Based on the molecular weight (39,780 Da) and length of the RNA standard (117 bp), the copy number was calculated. A series of the obtained RNA standards with 10-fold dilutions was used to establish the standard curve for qRT-PCR. qRT-PCR was performed in 8-well strip tubes in triplicate using the Light Cycler 480 PCR System (Roche). The same PCR conditions and system were used for each target, according to the manufacturer’s protocol (Takara Bio Inc. Dalian, RR086A). Known positive and negative controls were used throughout, and all assays were performed in triplicate.

**Table 1:** The special primers for atypical porcine pestivirus (APPV) used in the study.

Primer	Sequence (5′–3′)	Target	Purpose
APPV_NS3-F	CAGAGRAAAGGKCGAGTGGG	NS3	qRT-PCR
APPV_NS3-R	ACCATAYTCTTGGGCCTGSAG	NS3	qRT-PCR
APPV_NS3-T7-F	GATCACTAATACGACTCACTATAGGG CAGAGGAAAGGCCGAGTGGG	NS3	qRT-PCR
APPV_NS5B-F	CCTGGGACACTCAAGTAACGA	NS5B	PCR
APPV_NS5B-R	GTGTCCCTTTGTTAG CTGCAT	NS5B	PCR


### Determination of Nucleotide Sequences

Based on the APPV/GER/01 sequence, primers targeting various fragments of the full-length gene were designed (primer sequences designed in-house and available on request). cDNA from an APPV-positive porcine serum sample was used as a template for PCR. The amplified products were purified with a gel purification kit (OMEGA Bio Inc. USA, D2500-02) following the manufacturer’s instructions. Sequencing was performed using an ABI Prism 3730XL genetic analyzer (Applied Biosystems) by Shanghai Sangon Biotechnology Co., Ltd. The nucleotide sequences were edited using the Seqman module of the DNAStar package. The sequence of APPV_GD is available in GenBank with the accession number: KY624591.

### Nucleotide Identity and Phylogenetic Analysis

The pestivirus sequences used in this article were downloaded from NCBI nucleotide databases^1^. Nucleotide identity analysis was performed using the MegAlign module of the DNAStar package. Phylogenetic analysis of the complete sequence was performed in MEGA 6.06. Sequences were aligned by CLUSTAL W. Phylogenetic trees were generated using neighbor-joining analysis. The bootstrap value was calculated using 1,000 replicates of the alignment.

### Origin and Dissemination Analysis

Partial sequences of the APPV NS5B gene (528 bp) and associated metadata were retrieved from the NCBI nucleotide database. As APPV is a newly discovered virus, the limited dataset consisted of 25 sequences isolated from four countries: Germany, the Netherlands, USA, and China (shown in Supplementary Table 1). CLUSTAL W was used for sequence alignment. jModelTest was used for selecting the best-fit nucleotide substitution model (TN93+γ) under the Akaike and Bayesian information criteria (Posada, 2008). A Bayesian Markov chain Monte Carlo approach, which was implemented using the BEAUti/BEAST package v1.7.5, was used for inferring the geographical origin and the global spatial dynamics of APPV (Drummond et al., 2012). To ensure effective sample sizes greater than 200 for each sampled parameter, four independent Markov chain Monte Carlo processes were run for 1 × 10^8^ generations. The first 10% steps were removed as burn-in for combined chains.

## Results

### Brief Survey of CT in China

To characterize the situation of CT in China, a brief survey was performed by visiting and investigating large-scale pig farms in Guangdong provinces, and consulting with the veterinarians in other provinces of China. CT was observed mainly in the south of China, especially in Sichuan and Guangdong provinces. The disease occurred intermittently throughout the year. CT-infected piglets were mostly produced by gilts (female pigs in their first pregnancy). A prevalence of 1%–2% was found in most CT-affected pig farms. Higher prevalence was observed in several farms, and one of the farms located in Guangdong had a prevalence of approximately 20%. All infected piglets were eliminated.

### Identification of APPV in Newborn Piglets with CT and Fattening Pigs and Sows

To detect the presence of APPV, serum samples were collected from three piglets affected by CT. The specific primers designed for NS5B amplification were used for PCR. The PCR products were subjected to agarose gel electrophoresis and sequencing. The gene fragment of APPV was detected in serum samples from all three piglets. Then, specific primers targeting different fragments of the full-length gene were designed. One of the serum samples was then used to determine the complete polyprotein-coding sequence. Finally, the complete polyprotein-coding sequence of the first Asian APPV strain, encoding 3,635 amino acids, was assembled. It was termed APPV_GD.

In total, 135 serum samples from fattening pigs and sows at 10 farms were screened. PCR-based detection showed that APPV genomes were present in seven samples from five farms. A total APPV prevalence of 5.2% was calculated (**Table 2**). Even though the background information (age, weight, and health of pigs) for these serum samples was not very clear or detailed, a high prevalence of APPV was shown.

**Table 2:** PCR results for piglet serum samples from 10 farms in China.

Farm	No. positive/Total	Percent (%)
1	1/5	20
2	0/12	0
3	0/5	0
4	0/15	0
5	2/15	13.3
6	1/6	16.7
7	1/13	7.7
8	0/16	0
9	0/24	0
10	2/24	8.3


### Tissue Tropism of APPV

**FIGURE 1:**
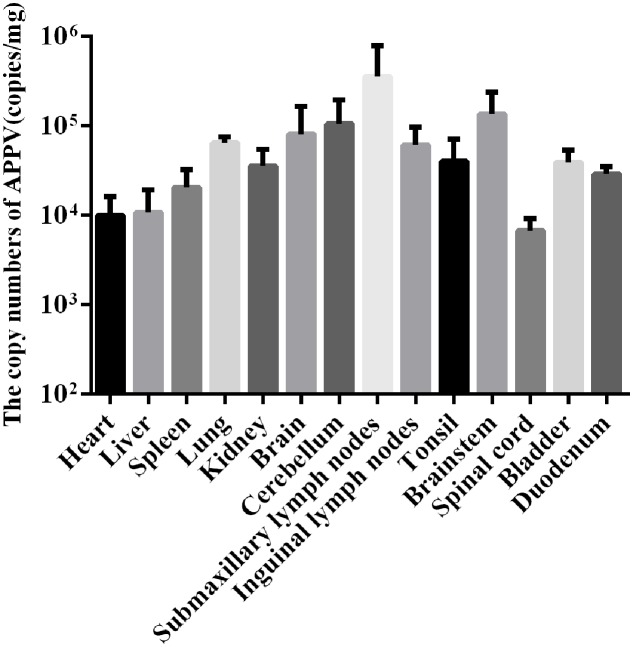
**Detection the copy numbers of APPV in different tissues of CT-affected piglets by qRT-PCR.** For the graph, the bars indicate the mean ± standard deviation (SD) values of all *n* = 3 independent experiments.

To determine the tissue tropism of APPV, 14 organ samples from the affected piglet were analyzed by qRT-PCR (**Figure 1**). Results suggested that viral genomes can be detected in all organ samples. Among them, the highest number of genomes was found in the submaxillary lymph nodes (3.56 ± 3.50 × 10^5^copies/mg), which is a peripheral lymphoid organ. The other peripheral lymphoid organs also contained a large number of APPV genomes, including the spleen (2.06 ± 0.95 × 10^4^copies/mg), tonsils (4.00 ± 2.53 × 10^4^copies/mg), and inguinal lymph nodes (6.09 ± 2.92 × 10^4^copies/mg). Interestingly, a large number of APPV genomes was also found in the nervous system, e.g., in the brainstem (1.35 ± 0.83 × 10^5^copies/mg), brain (8.11 ± 6.81 × 10^4^copies/mg) and cerebellum (1.05 ± 0.73 × 10^5^copies/mg). In addition, viral genomes were detected in the duodenum (2.90 ± 0.50 × 10^4^ copies/mg), which is part of the digestive system.

### Nucleotide Identity and Phylogenetic Analysis of APPV

**FIGURE 2:**
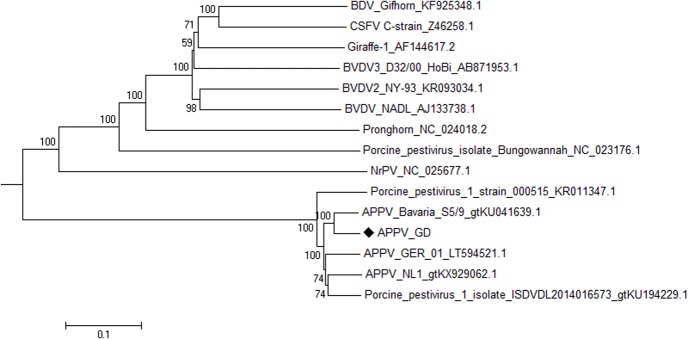
**Phylogenetic trees were generated by the distance-based neighbor-joining method using MEGA 6.06 software based on the full-length sequences.** The sequence marked with “
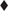
” is the APPV (APPV_GD) sequenced in this study.

To examine the molecular characteristics of the first Asian APPV genome, nucleotide sequence identity analysis was performed using representative pestivirus sequences. APPV_GD was found to share the highest nucleotide sequence identity with a German APPV, namely APPV_Bavaria_S5/9, with 93.5% nucleotide sequence identity. With the other four APPV sequences, 88.3%–91.1% nucleotide sequence identity was found. Remarkably, the partial sequence of RaPV, a pestivirus isolated from a bat (Rhinolophus affinis), exhibited 64.5% nucleotide sequence identity with APPV_GD. APPV_GD showed 41.1%–42.7% nucleotide identity with other pestiviruses, as shown in **Table 3**. Phylogenetic analysis of the complete polyprotein sequence of APPV_GD indicated that the newly discovered virus was in the atypical pestivirus lineage, with distant relatedness to classical pestiviruses such as classical swine fever virus (CSFV), bovine viral diarrhea virus (BVDV), and border disease virus (BDV) (**Figure 2**).

**Table 3:** Nucleotide identity of APPV_GD to related pestiviruses with sequences in GenBank.

No.	Virus	Identity (%)
1	APPV_Bavaria_S5/9_gtKU041639.1	93.5
2	APPV_GER_01_LT594521.1	91.1
3	APPV_NL1_gtKX929062.1	91.1
4	Porcine_pestivirus_1_isolate_ISDVDL2014016573_gtKU194229.1	90.7
5	Porcine_pestivirus_1_strain_000515_KR011347.1	88.3
6	Rhinolophus affinis pestivirus 1_ JQ814854.1	64.5
7	BVDV_NADL_AJ133738.1	42.7
8	BDV_Gifhorn_KF925348.1	42.5
9	BVDV3_D32/00_HoBi_AB871953.1	42.5
10	CSFV_C-strain_Z46258.1	42.3
11	Giraffe-1_AF144617.2	42.3
12	BVDV2_NY-93_KR093034.1	42.1
13	NrPV_NC_025677.1	41.2
14	Porcine_pestivirus_isolate_Bungowannah_NC_023176.1	41.1
15	Pronghorn_NC_024018.2	41.0


### Origin and Dissemination Analysis of APPV

Based on a Bayesian analysis, the origin and dissemination of current APPV strains was jointly inferred (Bielejec et al., 2011). The analysis suggested that the tMRCA of current APPV strains emerged in Germany, and three major ancestral clades of the current APPV strains were diversified and spread worldwide. The results suggested that the first APPV migration event might have spread from Germany to the United States. Another migratory event was the spread from Germany to the Netherlands, followed by spread from Germany to China and possibly transmission back from America back to Germany.

## Discussion

As they are highly variable RNA viruses, the number of pestiviruses increases constantly. Several novel pestiviruses have been identified in various domestic and wild ruminant species in the last few decades (Kirkland et al., 2007; Wu et al., 2012; Firth et al., 2014). CT was first recorded nearly 100 years ago. Previous studies have suggested that the causes of CT are diverse and include malnutrition, heredity, and viral infections (Knox et al., 1978; Vannier et al., 1981; Bradley et al., 1983). However, the disease was largely attributed to an unidentified virus (Arruda et al., 2016). Innovations in sequencing technology led to the first description of APPV from porcine serum, using metagenomic sequencing in 2015 (Hause et al., 2015). Despite being a newly discovered pestivirus, APPV has shown a close association with the occurrence of CT in newborn piglets (Arruda et al., 2016; de Groof et al., 2016; Postel et al., 2016).

China has a huge pig industry, and a CT prevalence of about 1%–2% has been found in affected farms. Owing to the size of the pig industry and the 100% elimination rate, CT has caused extensive economic losses. In the present study, APPV was detected in all of the CT-affected piglets from two farms in Southern China. This result, which confirmed the close relationship between CT and APPV, was consistent with findings of Arruda et al. (2016), de Groof et al. (2016), Postel et al. (2016), and Schwarz et al. (2017). In total, 135 serum samples from sows and fattening pigs from 10 farms were screened. APPV was detected in 5 of the 10 farms sampled, and the APPV prevalence in these samples was approximately 5.2% (**Table 2**). This unexpectedly high prevalence of APPV suggests that the virus may be prevalent in most parts of Southern China. Testing with a larger number of farms over a wider geographical area is necessary in the future.

To further establish that APPV causes CT solely in newborn piglets, isolation of the virus was performed. We inoculated swine kidney cell lines (PK15, SK6, and IBRS) and a swine testis cell line (ST) with serum or organ homogenates from APPV-positive piglets, but could not detect the replication of APPV in any of these cell lines. Hause et al. (2015), Arruda et al. (2016), and de Groof et al. (2016) also previously attempted, without success, to isolate the virus through similar approaches. Beer et al. (2016) reported that APPV is able to replicate in a porcine kidney cell line (SPEV, cell line 0008, Collection of Cell Lines in Veterinary Medicine, FLI), but that further identification during passaging is necessary. Schwarz et al. (2017) reported that APPV could be isolated and passaged on different porcine cells, but showed very low titers. Thus, animal experiments involving controlled infection using pure virus stocks after culturing have not been performed to date. The CT reproduction studies by Arruda et al. (2016) and de Groof et al. (2016) were both achieved with serum samples containing APPV.

Tissue tropism of APPV was examined by qRT-PCR. A high number of APPV genomes were found in the central nervous system and peripheral lymphoid organs, potentially explaining the intense clinical neurological symptoms caused by this virus. APPV was additionally detected in other tissues, suggesting that it replicates systemically.

Phylogenetic analysis of the complete polyprotein sequences of known pestiviruses showed that APPV_GD belonged to a new lineage along with the other recently discovered APPV strain, and indicated a distant relatedness of these APPV strains to classical pestiviruses. The data strongly suggest that APPV represents a previously unrecognized pestivirus. Nucleotide identity analysis showed that APPV_GD shared the highest nucleotide sequence identity with a European APPV strain, APPV_Bavaria_S5/9, which was isolated in Germany. Coincidently, the present data showed that APPV_GD might also have originated in Germany, and not in America, which was the first location where APPV genome was assembled (Hause et al., 2015). However, limited data on APPV sequences are currently available; therefore, it is possible that the conclusions about the origin and dissemination of the virus could change when more APPV strains are isolated in the future.

In summary, our study reports the first description of APPV in China and establishes a quantitative PCR method for the detection of APPV. Meanwhile, we provide useful bioinformatics analyses of current APPV strains. Further studies should focus on virus isolation aimed at elucidating the mechanisms underlying pathogenesis and control virus transmission.

## Author Contributions

JY designed and performed experiments, purchased materials, and drafted the manuscript. ZH performed sequence analysis and software installation. JL, YH, JY, and HD completed the collection of samples. YZ, MZ, YZ, and JL helped with sample detection. JC and MZ conceived of the study, and participated in its design and coordination. All authors read and approved the final manuscript.

## Conflict of Interest Statement

The authors declare that the research was conducted in the absence of any commercial or financial relationships that could be construed as a potential conflict of interest.

## Footnotes

1 http://www.ncbi.nlm.nih.gov
