# Supplementary material for: Atypical Porcine Pestivirus as a Novel Type of Pestivirus in Pigs in China
Source: Front Microbiol. 2017 May 11;8:862. doi: 10.3389/fmicb.2017.00862 (PMC5425480; doi:10.3389/fmicb.2017.00862)
Supplement: Supplementary file 1 [file Table_1.DOCX]

**Supplementary Table**

Table 1. The Accession numbers of all 25 sequences of NS5B used in the study

| No. | Virus | Accession no. |
| --- | --- | --- |
| 1 | Atypical porcine pestivirus 1 isolate Bavaria S5/9 polyprotein gene, complete cds | NC_030653.1 |
| 2 | Atypical porcine pestivirus 1 isolate GD polyprotein gene, complete cds | KY624591 |
| 3 | Atypical porcine pestivirus isolate NL1 Farm1 polyprotein gene, complete cds | KX929062.1 |
| 4 | Porcine_pestivirus_1_isolate_ISDVDL2014016573 | KU194229.1 |
| 5 | Porcine_pestivirus_1_strain_000515 | KR011347.1 |
| 6 | Porcine pestivirus 1 isolate APPV_GER_01 genome assembly, complete genome | LT594521.1 |
| 7 | Porcine pestivirus 1 partial gene for NS5B, strain APPV_GER_2 | LT600759.1 |
| 8 | Porcine pestivirus 1 partial gene for NS5B, strain APPV_GER_3 | LT600760.1 |
| 9 | Porcine pestivirus 1 partial gene for NS5B, strain APPV_GER_5 | LT600762.1 |
| 10 | Porcine pestivirus 1 partial gene for NS5B, strain APPV_GER_6 | LT600763.1 |
| 11 | Porcine pestivirus 1 partial gene for NS5B, strain APPV_GER_7 | LT600764.1 |
| 12 | Porcine pestivirus 1 partial gene for NS5B, strain APPV_GER_8 | LT600765.1 |
| 13 | Porcine pestivirus 1 partial gene for NS5B, strain APPV_GER_9 | LT600766.1 |
| 14 | Porcine pestivirus 1 partial gene for NS5B, strain APPV_GER_10 | LT600767.1 |
| 15 | Porcine pestivirus 1 partial gene for NS5B, strain APPV_GER_11 | LT600768.1 |
| 16 | Porcine pestivirus 1 partial gene for NS5B, strain APPV_GER_12 | LT600769.1 |
| 17 | Porcine pestivirus 1 partial gene for NS5B, strain APPV_GER_13 | LT600770.1 |
| 18 | Porcine pestivirus 1 partial gene for NS5B, strain APPV_GER_14 | LT600771.1 |
| 19 | Porcine pestivirus 1 partial gene for NS5B, strain APPV_GER_15 | LT600772.1 |
| 20 | Porcine pestivirus 1 partial gene for NS5B, strain APPV_GER_16 | LT600773.1 |
| 21 | Porcine pestivirus 1 partial gene for NS5B, strain APPV_GER_17 | LT600774.1 |
| 22 | Porcine pestivirus 1 partial gene for NS5B, strain APPV_GER_18 | LT600775.1 |
| 23 | Porcine pestivirus 1 partial gene for NS5B, strain APPV_GER_19 | LT600776.1 |
| 24 | Porcine pestivirus 1 partial gene for NS5B, strain APPV_GER_20 | LT600777.1 |
| 25 | Porcine pestivirus 1 partial gene for NS5B, strain APPV_GER_21 | LT600778.1 |
